# Identification of diagnostic markers for major depressive disorder by cross-validation of data from whole blood samples

**DOI:** 10.7717/peerj.7171

**Published:** 2019-06-21

**Authors:** Huimei Wang, Mingwei Zhang, Qiqi Xie, Jin Yu, Yan Qi, Qiuyuan Yue

**Affiliations:** 1 Department of Integrative Medicine and Neurobiology, State Key Laboratory of Medical Neurobiology, Institute of Brain Science, School of Basic Medical Sciences, Shanghai Medical College, Fudan University, Shanghai, China; 2 Department of Radiation Oncology, First Affiliated Hospital of Fujian Medical University, Fujian, Fuzhou, China; 3 Department of Orthopaedics, Second Hospital of Lanzhou University, Lanzhou, Gansu, China; 4 Yunnan Provincial Key Laboratory of Traditional Chinese Medicine Clinical Research, First Affiliated Hospital of Yunnan University of Traditional Chinese Medicine, Yunnan, Kunming, China; 5 Department of Radiology, Fujian Cancer Hospital & Fujian Medical University Cancer Hospital, Fujian, Fuzhou, China

**Keywords:** Differentially expressed gene, Major depressive disorder, Inflammation, Correlation network analysis, Mitochondrial dysfunction, Diagnostic value

## Abstract

**Background:**

Major depressive disorder (MDD) is a severe disease characterized by multiple pathological changes. However, there are no reliable diagnostic biomarkers for MDD. The aim of the current study was to investigate the gene network and biomarkers underlying the pathophysiology of MDD.

**Methods:**

In this study, we conducted a comprehensive analysis of the mRNA expression profile of MDD using data from Gene Expression Omnibus (GEO). The MDD dataset (GSE98793) with 128 MDD and 64 control whole blood samples was divided randomly into two non-overlapping groups for cross-validated differential gene expression analysis. The gene ontology (GO) enrichment and gene set enrichment analysis (GSEA) were performed for annotation, visualization, and integrated discovery. Protein–protein interaction (PPI) network was constructed by STRING database and hub genes were identified by the CytoHubba plugin. The gene expression difference and the functional similarity of hub genes were investigated for further gene expression and function exploration. Moreover, the receiver operating characteristic curve was performed to verify the diagnostic value of the hub genes.

**Results:**

We identified 761 differentially expressed genes closely related to MDD. The Venn diagram and GO analyses indicated that changes in MDD are mainly enriched in ribonucleoprotein complex biogenesis, antigen receptor-mediated signaling pathway, catalytic activity (acting on RNA), structural constituent of ribosome, mitochondrial matrix, and mitochondrial protein complex. The GSEA suggested that tumor necrosis factor signaling pathway, Toll-like receptor signaling pathway, apoptosis pathway, and NF-kappa B signaling pathway are all crucial in the development of MDD. A total of 20 hub genes were selected via the PPI network. Additionally, the identified hub genes were downregulated and show high functional similarity and diagnostic value in MDD.

**Conclusions:**

Our findings may provide novel insight into the functional characteristics of MDD through integrative analysis of GEO data, and suggest potential biomarkers and therapeutic targets for MDD.

## Introduction

The prevalence and incidence of major depressive disorder (MDD), which is ranked as the leading cause of the global disease burden and death by suicide (Ferrari et al., 2013; Hasin et al., 2018), are continuously increasing. MDD is a severe, recurrent, and debilitating disease characterized clinically by a multifactorial and multistage process (mild, moderate, or severe depression) associated with the interaction between genetic and environmental factors. Moreover, the duration, number, and pattern of episodes of MDD are variable, the term of “recovery” is used to describe patients that have regained their usual function and are no longer symptomatic after an episode of MDD in community settings. With timely and appropriate treatment, episodes last approximately 3–6 months, and most patients recover within 12 months (Lépine & Briley, 2011; Malhi & Mann, 2018). On the contrary, the probability of recurrence increases and the outcome is less favorable in longer-term episodes, and the recovery rate drops to approximately 60%, 40%, and 30% at 2, 4, and 6 years, respectively, with comorbid anxiety having an important role in limiting recovery (Malhi & Mann, 2018). It should be noted that early diagnosis and treatment would unquestionably decrease the morbidity and mortality associated with depression. A biomarkers is a measurable indicator of some biological condition or state. Identification of biomarkers would be a key step for MDD. C-reactive protein, an acute-phase protein, is widely used as biomarker in MDD and inflammation (Chamberlain et al., 2019). Recently, microRNAs and exosomes have been applied as diagnostic and therapeutic biomarkers in MDD patients (Tavakolizadeh et al., 2018). In addition, an interesting study identifies distinct “biotypes” of depression using fMRI, which could be diagnostic biomarkers and may predict treatment response (Wager & Woo, 2017). However, the accuracy of biomarkers for diagnosis and prognosis of MDD is still largely limited because the pathogenesis of depression is complex and heterogeneous. Thus, investigation of the molecular mechanisms underlying MDD is crucial, and may contribute to identification of the precise targets and essential biomarkers for MDD diagnosis.

A variety of differential diagnostic criteria are associated with MDD. The clinical standardized definitions, such as those provided by the Diagnostic and Statistical Manual of Mental Disorders, Fourth Edition, Text Revision; Hamilton Rating Scale for Depression (HAMD); and Montgomery–Asberg Depression Rating Scale (Nemeroff, 2007), are the most common classical methods for determining MDD, and can be applied either in the clinic or in treatment trials. For example, HAMD, a 17-item instrument, is used to examine the intensity and frequency of depression severity in individuals with MDD. Scores on HAMD represents the severity ranges of MDD: normal (0–7); mild depression (8–16); moderate depression (17–23); and severe depression (≥24). A potential problem of using threshold scores for identification and classification is low-accuracy in distinguishing the depression severity and prognoses across cases (Fitzgerald et al., 2018). Empirical methods such as positron emission tomography and functional magnetic resonance imaging have contributed to identification of the brain regions that are affected in MDD (Siegle, Carter & Thase, 2006). A challenge in these studies is to disentangle the different contribution of depression and other comorbidities to the overall clinical picture. Mostly, an accurate diagnosis can be achieved via detailed history-taking, mental status and physical examination, and laboratory tests. Notably, an emerging and powerful method for investigating the pathogenesis of this disorder is examining peripheral blood for verification of gene expression levels (Hepgul et al., 2013). Studies employing these peripheral blood examinations have analyzed biomarkers via relatively accessible and low-invasiveness procedures, and demonstrated that peripheral inflammation precedes the emergence of symptoms in patients with depression (Khandaker et al., 2014). Moreover, the differential expression of genes in venous blood samples can be measured using flow cytometry and polymerase chain reaction. These approaches have relatively low effectiveness and throughput, which can now be improved using microarray-based technologies for high-throughput functional genomic discovery. Microarray is a promising and popular method for large-scale gene expression profiling, greatly facilitating the analysis of thousands of mRNAs simultaneously in a single experiment.

Several studies have been performed to improve the understanding of the molecular mechanisms underlying microarray analysis. A meta-analysis of genome-wide expression studies on MDD has been conducted using different microarray platforms and tissues, such as blood, the amygdala, and the prefrontal cortex (Forero, Guio-Vega & González-Giraldo, 2017). However, multi-platform analyses, the use of various tissues, and a lack of important variables, such as postmortem intervals or severity of the disorder, in the raw data contribute to inevitable batch effects. Moreover, a previous study investigated differentially expressed genes (DEGs, a group of genes that differentially express in different experimental conditions) in peripheral blood samples from 38 patients with MDD and 14 healthy controls (Woo et al., 2018), but the small sample size diminished the reliability of the results. A more careful examination was performed in two case-control studies of MDD using microarray data from whole blood samples (GSE98793) to investigate changes in peripheral inflammation (Leday et al., 2018). In particular, they investigated DEGs focusing on the changes in innate and adaptive immune gene expression by comparing 113 patients with MDD (57 comorbid with anxiety disorder, 56 without anxiety) and 57 healthy controls. Moreover, although a series of bioinformatics analyses has thoroughly investigated the potential biomarkers of immunological stratification in patients with MDD, it remains to be examined how functional systems and molecules other than immunological biomarkers affect the pathophysiology of MDD. Thus, further analyses are warranted to identify more robust and reliable diagnostic biomarkers, with cross-validation, large samples to comprehensively consider the abnormalities in the molecular mechanisms involved in MDD.

Hence, the aim of this study was to identify potential diagnostic biomarkers and biological functions related to MDD from the Gene Expression Omnibus (GEO; Edgar, Domrachev & Lash, 2002). Further, DEGs were investigated to distinguish patients with MDD from healthy controls via cross-validation. Moreover, the biological processes (BPs) involved were analyzed using gene ontology (GO) enrichment and gene set enrichment analysis (GSEA) pathways for the DEGs. In addition, the top 20 hub genes screened via protein–protein interaction (PPI) network were selected for their functional similarity, and their diagnostic value was assessed. Our study may provide some insights into the molecular mechanisms underlying MDD based on its pathophysiology.

## Materials and Methods

### Data collection and preprocessing

**Figure 1:**
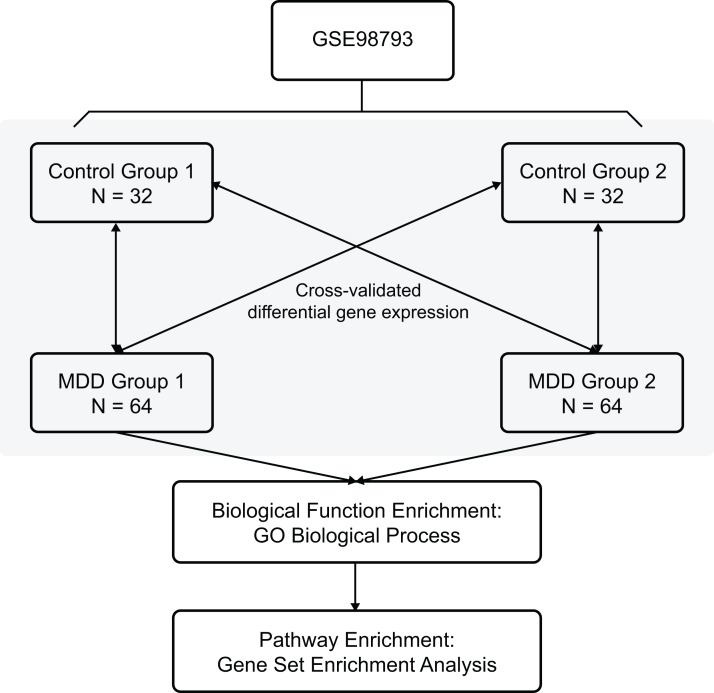
Flow chart of methodologies applied in the current study.

The Gene Expression Omnibus database is an international public repository which archives and distributes high-throughput gene expression and genomics data sets. The gene expression dataset GSE98793 (Leday et al., 2018) was downloaded from the GEO database (GPL570 (HG-U133_Plus_2) Affymetrix Human Genome U133 Plus 2.0 Array) and annotated in the R software (R Foundation for Statistical Computing, Vienna, Austria) using annotation files. The species selected was *Homo sapiens*, and the data type was microarray expression profiles. The whole blood samples included 128 MDD (diagnosed post hoc by the Mini-International Neuropsychiatric Interview) and 64 control healthy samples. Clinical and demographic characteristics of the MDD patients are shown in Table 1. The MDD and control blood samples were divided randomly into two non-overlapping groups each (MDD group 1, MDD group 2, control group 1, and control group 2) for cross-validation. The overview of the workflow is shown in Fig. 1.

**Table 1:** Clinical and demographic characteristics of the MDD patients.

	All subjects	MDD	Controls
Patients	192	128	64
Gender			
Male	48	32	16
Female	144	96	48
Comorbidities			
Anxiety	64	64	0
Without anxiety	64	64	0
Age (years)	52 ± 1	52 ± 1	52 ± 1

### Screening of DEGs

The Affy (Gautier et al., 2004) and limma (Smyth, 2005) packages were applied to the microarray data to filter the DEGs by comparing both MDD groups to both healthy control groups using a multivariate linear model using moderated *t*-statistic. Data were corrected for multiple comparisons using false discovery rate adjustment, and genes with |logFC| (an absolute log2 value in the fold change of the expression of the genes) >0.6 and *P*-value < 0.05 (Dalman et al., 2012) in all four comparisons were identified as DEGs.

### Functional and pathway enrichment analysis

The GO analysis serves as a bioinformatics tool that provides structured annotations, including BPs, molecular functions (MFs), and cellular components (CCs), for genes and gene products. Modules related to biological function were investigated using UpSetR (Conway, Lex & Gehlenborg, 2017) to determine the functional and pathway enrichment for BPs in GO. Functional and pathway enrichment were analyzed using hypergeometric test and Bonferroni correction. We also used GSEA which is a statistical approach for determining whether the genes from particular pathways or other predefined gene sets are differentially expressed in different phenotypes (Subramanian et al., 2005). Reactome pathways were analyzed with GSEA, using clusterProfiler (Yu et al., 2012) to define every functional cluster. C2.all.v6.2.symbols.gmt was selected as the reference gene set. False discovery rate <0.1, and *P*-value < 0.01 were set as the cut-off criteria.

### PPI network construction

The PPI information available in the STRING network in the STRING database (http://string-db.org, version 10) (Szklarczyk et al., 2015) is useful for predicting physical and functional interactions. All DEGs were mapped to the STRING database, and the interactions with reliability scores more than 0.4 were selected to analyze the relationship of the DEGs. Cytoscape (Shannon et al., 2003) was used to select the key nodes with the strongest connectivity for visualizing molecular interaction networks. CytoHubba, a Cytoscape plugin, was used to identify the top 20 hub genes of the merged network (Chin et al., 2014). NetworkAnalyst (https://www.networkanalyst.ca/faces/home.xhtml) (Xia, Gill & Hancock, 2015) is a visual analytics platform for PPI networks. We inputted the 20 hub genes into NetworkAnalyst for visualization of PPI networks. The expression analysis of the top 20 hub genes with the highest ranking are shown in Table 2.

**Table 2:** The expression analysis of the top 20 hub genes with the highest ranking.

Gene symbol	Entrez ID	logFC	*P*-value	Score
*MRPS11*	64963	−0.698	6.49E-24	8.92E+13
*MRPS2*	51116	−0.502	6.07E-13	8.90E+13
*MRPL2*	51069	−1.105	4.37E-25	8.64E+13
*MRPL15*	29088	−0.823	3.60E-18	8.37E+13
*MRPL16*	54948	−0.828	8.73E-23	8.37E+13
*MRPS7*	51081	−0.905	5.15E-21	6.55E+13
*MRPS18*	6222	−0.835	1.15E-19	4.74E+13
*RPS3*	6188	−0.778	6.64E-21	4.74E+13
*RPL11*	6135	−0.927	1.14E-20	4.74E+13
*RPL26L1*	51121	−0.868	2.30E-21	4.73E+13
*RPL6*	6128	−0.689	1.1E-20	4.73E+13
*RPL19*	6143	−0.732	6.99E-24	4.73E+13
*RPS19*	6223	−0.714	1.01E-20	4.73E+13
*NAS2*	10412	−0.962	7.7E-23	4.73E+13
*NHP2*	55651	−1.344	3.1E-25	4.73E+13
*RPP38*	10557	−0.941	8.2E-23	4.73E+13
*RPL29*	6159	−1.065	7.18E-22	4.73E+13
*MRPL36*	64979	−0.757	4.01E-18	4.73E+13
*MRPL27*	51264	−1.257	1.84E-23	4.73E+13
*MRPL9*	65005	−0.752	9.09E-22	4.73E+13

### Distributions of hub genes

The distributions of all DEGs in GSE98793 were identified. Moreover, the functional similarity among proteins was evaluated using the geometric mean of semantic similarities in CCs and MFs through the GOSemSim package (Yu et al., 2010).

### Setting the cut-off score based on receiver operating characteristic curve analysis

Receiver operating characteristic (ROC) curve analysis, which yields indictors of accuracy such as the area under the curve (AUC), provides the basic principle and rationale for distinguishing between the specificity and sensitivity of diagnostic performance (Akobeng, 2007). The maximum value of the sum of specificity and sensitivity was used as the cut-off score for each hub gene. The “pROC” package of the R software was applied for ROC curve analysis (Robin et al., 2011).

### Statistical analysis

All statistical analyses were performed as the means ± standard deviation. The R software (version 3.5.2) was utilized to measure the data. A *P*-value < 0.05 was considered statistically significant.

## Results

### Differentially expressed genes identification

**Figure 2:**
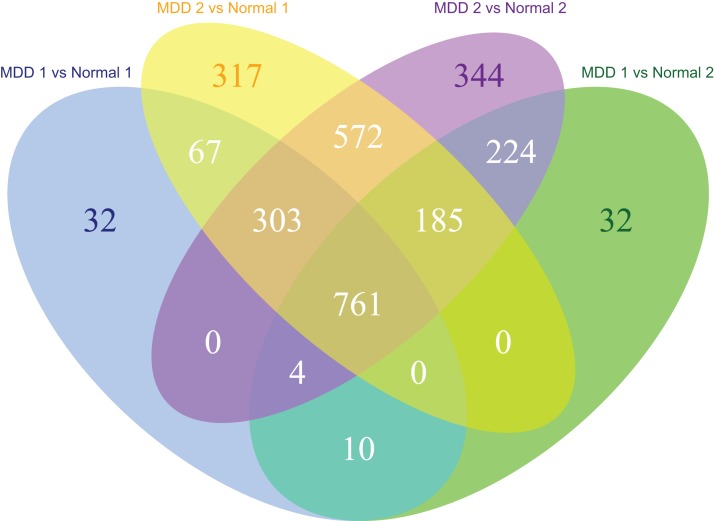
Venn diagram of the differentially expressed genes significantly associated with major depression disorder which were short-listed for the cross-validation.

The MDD and control blood samples were divided into two groups for cross-validation. A total of 64 samples remained in both MDD groups and 32 samples remained in both control groups. Cross-validation of the data from MDD and control groups identified 761 DEGs in the MDD groups (Fig. 2).

### GO enrichment analysis of DEGs

**Figure 3:**
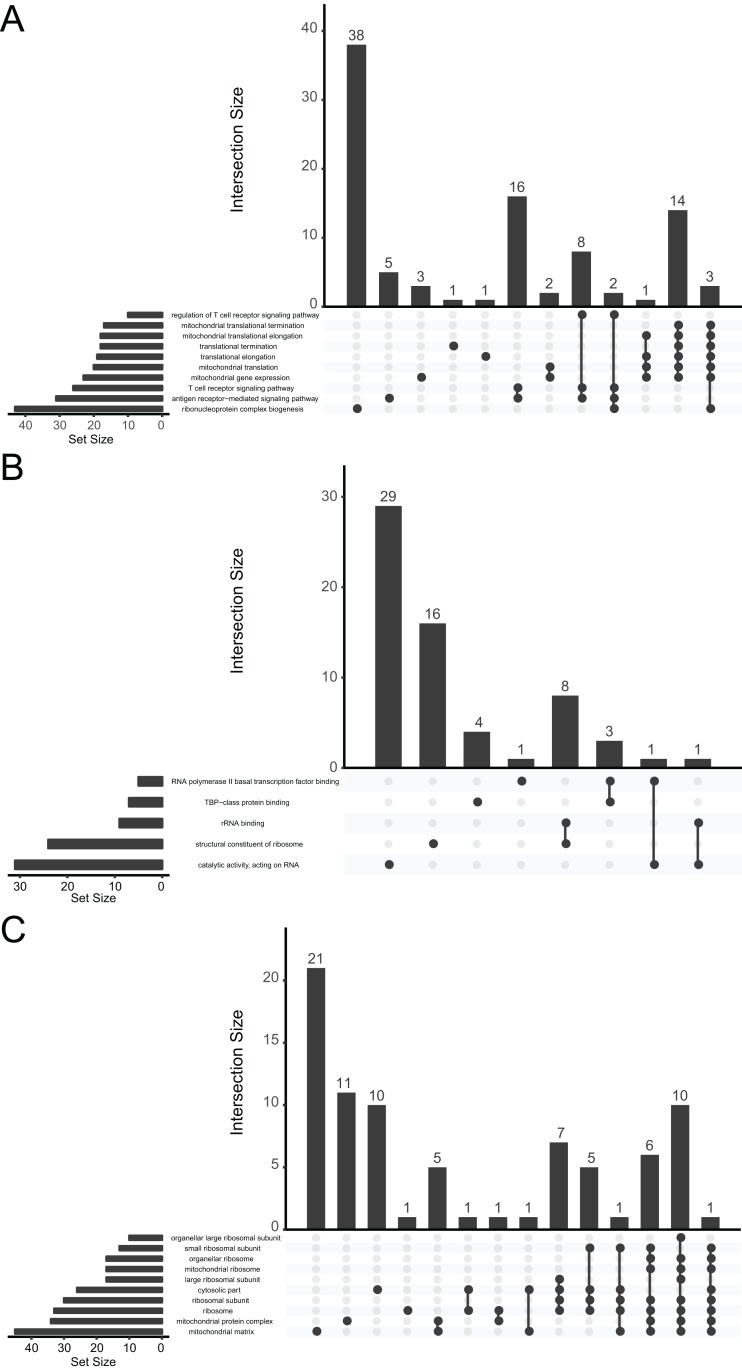
UpSetR plot demonstrating distribution of the gene ontology annotations for major depressive disorder in (A) biological processes, (B) molecular functions, and (C) cellular components.

We performed a functional enrichment analysis for further investigation of DEGs. The data indicated that the DEGs were significantly enriched in GO terms. The GO analysis demonstrated that changes in BPs were mainly enriched in ribonucleoprotein complex biogenesis, antigen receptor-mediated signaling pathway, T-cell receptor signaling pathway, mitochondrial gene expression, mitochondrial translation, and translational elongation (Fig. 3A). Changes in MFs were significantly enriched in catalytic activity (acting on RNA), structural constituent of ribosomes, rRNA binding, TATA-binding protein-class protein binding, and RNA polymerase II basal transcription factor binding (Fig. 3B). Changes in CCs for the DEGs were enriched mainly in the mitochondrial matrix, mitochondrial protein complex, ribosome, ribosomal subunit, cytosolic part, and large ribosomal subunit (Fig. 3C).

### GSEA of MDD-related genes

**Figure 4:**
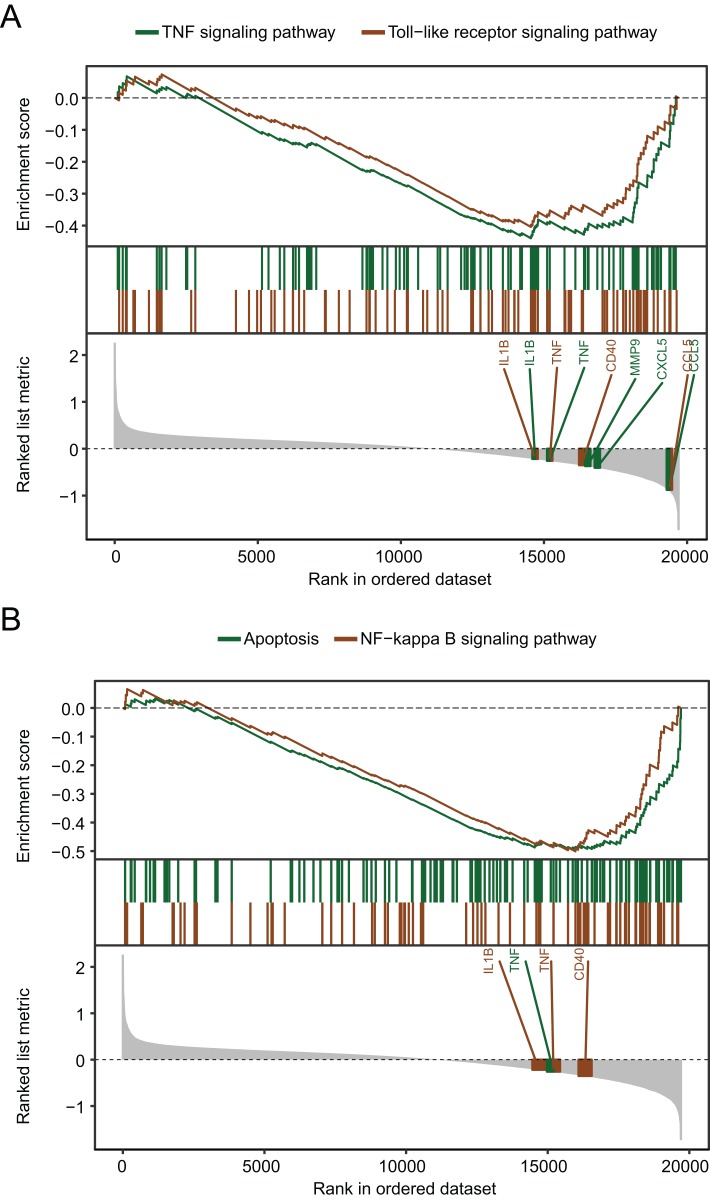
Gene set enrichment analysis of the gene expression profiles of the GSE98793 dataset. (A) Gene set enrichment analysis demonstrated that the TNF signaling pathway and the Toll-like receptor signaling pathway were enriched in MDD. (B) Gene set enrichment analysis demonstrated that apoptosis and the NF-kappa B signaling pathway were enriched in MDD.

The biological pathways that were significantly altered in MDD blood samples compared with the control blood samples were determined using GSEA. The GSEA of GSE98793 gene expression profiles suggested that MDD is mainly related to the apoptosis pathway, and the tumor necrosis factor (TNF), Toll-like receptor (Fig. 4A), and nuclear factor kappa-light-chain-enhancer of activated B cells (NF-κB) signaling pathways (Fig. 4B).

### PPI network analysis of DEGs

**Figure 5:**
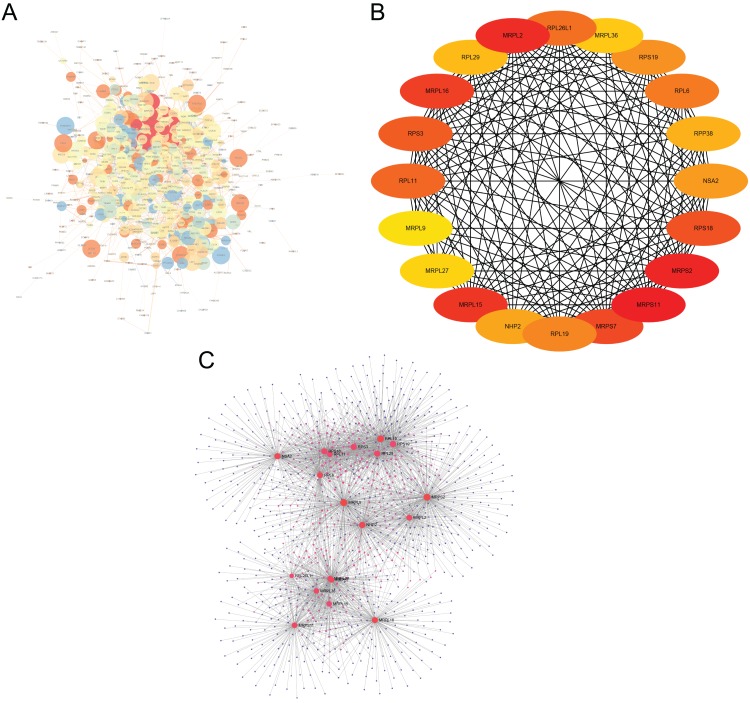
Major depressive disorder-specific network. (A) Protein–protein interaction network of differentially expressed genes using the STRING database. (B) The CytoHubba plugin was used to analyze the top 20 hub genes with maximum correlation criterion. (C) The hub genes with the top 20 scores were analyzed using the NetworkAnalyzer plugin.

The interactions of 761 DEGs were analyzed using the STRING online database to investigate the PPI network underlying MDD. The obtained results were analyzed using the Cytoscape software (Fig. 5A). The cytoHubba plugin was then used to investigate the top 20 hub genes associated with MDD (Fig. 5B). Moreover, the visualized network of the hub genes is shown using the NetworkAnalyzer online tool (Fig. 5C).

### Distributions of hub genes

**Figure 6:**
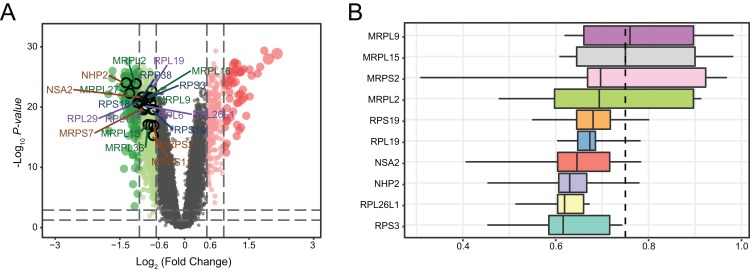
Genetic screening for hub genes in patients with major depressive disorder. (A) Volcano plot of fold changes in the expression of the hub genes. (B) Summary of functional similarities of the top 10 hub genes.

We determined the distributions of 761 DEGs from MDD and healthy control blood samples (Fig. 6A). Among all DEGs, the top 20 hub genes were identified as being downregulated in MDD. Notably, three hub genes with the highest ranking were found to be downregulated: mitochondrial ribosomal protein L2 (*MRPL2*), *NHP2*, and NOP-seven-associated 2 (*NSA2*). Moreover, we ranked top 10 genes among the 20 hub genes based on the average functional similarity (Fig. 6B). Mitochondrial ribosomal protein L9 (*MRPL9*), mitochondrial ribosomal protein L15 (*MRPL15*), and mitochondrial ribosomal protein S2 (*MRPS2*) were the top three proteins potentially playing key roles in MDD; *MRPL9* was the only protein with a cut-off value >0.75.

### Using hub genes for MDD diagnosis

**Figure 7:**
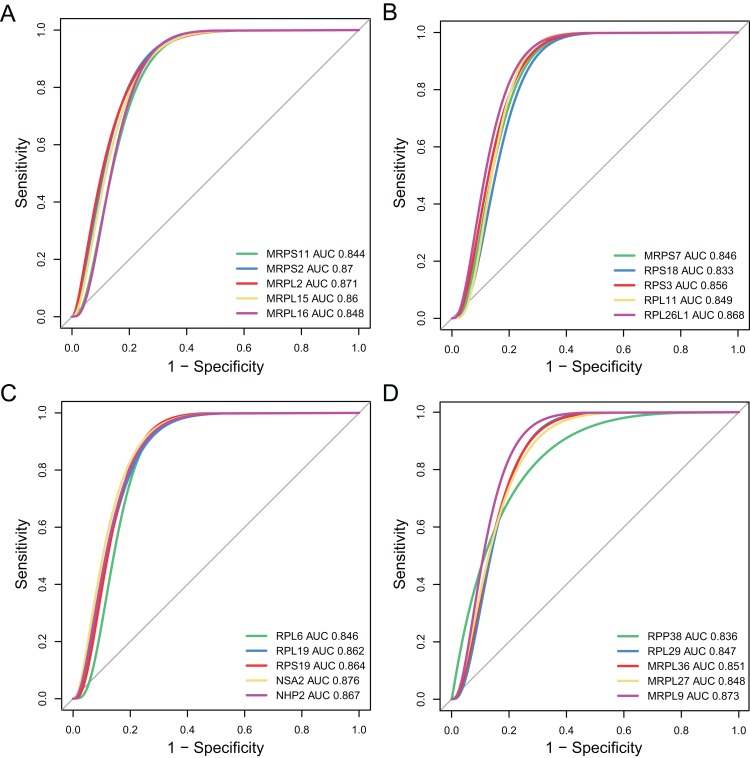
Validation of diagnostic value of the hub genes for major depressive disorder (MDD). (A–D) Receiver operating characteristic curve of the hub genes for diagnosis of MDD.

The diagnostic accuracy of the top 20 hub genes was assessed using ROC curve analysis (Fig. 7). The areas under the ROC curves were 0.844, 0.87, 0.871, 0.86, and 0.848 for *MRPS11*, *MRPS2*, *MRPL2*, *MRPL15*, and *MRPL16*, as shown in Fig. 7A. The areas under the ROC curves were 0.846, 0.833, 0.856, 0.849, and 0.868 for *MRPS7*, ribosomal protein S18 (*RPS18*), *RPS3*, ribosomal protein L11 (*RPL11*), and *RPL26L1*, as shown in Fig. 7B. The areas under the ROC curves were 0.846, 0.862, 0.864, 0.876, and 0.867 for *RPL6*, *RPL19*, *RPS19*, *NSA2*, and *NHP2*, as shown in Fig. 7C. The areas under the ROC curves were 0.836, 0.847, 0.851, 0.848, and 0.873 for *RPP38*, *RPL29*, *MRPL36*, *MRPL27*, and *MRPL9*, as shown in Fig. 7D.

## Discussion

Worldwide, MDD is a recurrent lifelong mental disorder of very high prevalence. The 12-month prevalence of MDD is approximately 6.6%, and the lifetime risk is 15–18% (Malhi & Mann, 2018). Increasing number of studies are being performed to develop a non-invasive and quantitative clinical test; however, no specific and sensitive biomarkers are available for the diagnosis and treatment of MDD yet. Therefore, in order to identify effective diagnostic biomarkers of MDD, we performed an integrated analysis on a large MDD cohort of 128 MDD patients and 64 healthy controls, using whole-genome microarray data for mRNA expression. A total of 761 DEGs were identified in the MDD group via cross-validation. Furthermore, GO enrichment analysis and GSEA showed that these enriched modules and pathways are closely related to the immune response and mitochondrial dysfunction observed in MDD. In addition, the top 20 hub genes associated with MDD, which were identified in the PPI network, showed high functional similarity and diagnostic values for MDD.

In the first part of the present study, we identified 761 DEGs in the GSE98793 dataset, collected from 128 MDD to 64 control whole blood samples, using cross-validation. To investigate the BPs of the DEGs involved in MDD, GO enrichment analyses were performed. Of the MF annotations, ribonucleoprotein complex biogenesis, antigen receptor-mediated signaling pathway, catalytic activity (acting on RNA), structural constituent of ribosomes, mitochondrial matrix, and mitochondrial protein complex were found to be significantly associated with the occurrence and development of MDD. Mitochondria play a critical role in the modulation of synaptic and neural plasticity required for the formation of novel neuronal synapses and pathways, as well as regulation of cellular Ca^2+^ homeostasis, oxidative stress, and apoptosis. Mitochondrial dysfunction has recently drawn considerable attention due to the postulation that impaired mitochondrial bioenergetics could be the basis for the pathophysiology of MDD through multiple potential pathways, including those related to genetics/genomics, oxidative stress, alterations in neuroplasticity, and inflammation (Klinedinst & Regenold, 2015). A previous study concluded that patients with mitochondrial disorders exhibit a higher rate of psychiatric illness than the general population; the authors reported that among 36 adults with mitochondrial cytopathies, the lifetime prevalence rate of psychiatric illnesses was up to 70% (Fattal et al., 2007). Taken together, these observations imply that mitochondrial dysfunction may be a major contributor to depression.

In the second part of the present study, in order to investigate the biological functions of the DEGs associated with MDD, GSEA was performed. The apoptosis pathway, and the TNF, Toll-like receptor, and NF-κB signaling pathways were the top four significantly enriched pathways. Interestingly, we noted that the most enriched pathway in our analysis was associated with immune response, inflammation, and apoptosis. Studies on rodent models and patients with depression show high levels of TNF-α, interleukin-1β, and interleukin-6, which are induced by infection, injury, and psychological stress (Haroon et al., 2018; Miller, Maletic & Raison, 2009; Wang et al., 2018). Consistent with our data, analysis of microarray results for MDD from other mRNA datasets also revealed that immune and inflammatory responses play a critical role in the regulation network of MDD (Woo et al., 2018). Additionally, it has been hypothesized that external stressors may induce depressive disorders via stimulation of inflammatory, oxidative, and apoptotic mechanisms, closely related to the pathways, such as TNF-α, NF-κB, Toll-like receptor, and apoptosis (Kubera et al., 2011). Our data-mining results further confirmed that inflammatory responses play a key role in the etiology of depression.

In the PPI network identified in the present study, 20 DEGs were highlighted as the most significant hub genes, with multiple interactions in the network. Further investigation of these genes may reveal the pathophysiology of MDD. All of these hub genes were identified as being significantly downregulated in MDD. Moreover, to identify the proteins involved in the pathophysiology of MDD, the top 10 genes were ranked among the 20 hub genes based on their average functional similarity. Moreover, MRPL9, MRPL15, and MRPS2 were ranked as the top three proteins potentially serving as central regulators in MDD. With regard to diagnostic value, the AUC of the 20 hub genes were analyzed. All the AUC values were in the range 0.830–0.900, suggesting that these genes possess moderate accuracy (Akobeng, 2007) in diagnostic examinations and may be promising targets for the diagnosis of MDD. Mitochondrial disorders may be induced by mutations in the mitochondrial and nuclear DNA contributing to impaired production of cellular energy (adenosine triphosphate) (Koene et al., 2009). *NSA2*, *MRPL9*, and *MRPL2* showed the three highest prognostic values among the hub genes. These genes encode mitochondrial and cytosolic ribosomal proteins, including MRPL, MRPS, RPL, and RPS, which play critical roles in translation, transcription, proliferation, and neural plasticity. NSA2, also known as tumor growth factor-β inducible nuclear protein 1, is predicted to serve as a cell cycle repressor and plays a crucial role in cell proliferation (Zhang et al., 2010). A previous study has demonstrated that NSA2 is activated after permanent middle cerebral occlusion in an Alzheimer’s disease mouse model (Tseveleki et al., 2010); NSA2 was predicted to be related to brain defense and tissue repair in the pathological process of Alzheimer’s disease. Similar decreased NSA2 may also be found in MDD. Consistent with our results, another RNA-sequencing study reported that the expression of multiple ribosomal genes, including RPL6 and RPL29, downregulation in the hypothalamus of male mice under chronic social defeat stress, which contributes to the development of depression- and anxiety-like symptoms (Smagin et al., 2016). In addition, mutations in mitochondrial or nuclear DNA have been implicated in a variety of neurological diseases, such as depression or personality disorder (McFarland, Taylor & Turnbull, 2010). Especially, MRPS15 (chromosome 1p34.3) is a clinical candidate for depressive syndrome (O’Brien, O’Brien & Norman, 2005). Notably, depressive behavior is associated with mitochondrial disorder in children (Morava et al., 2010), which suggests that the genes encoding cytosolic and mitochondrial ribosomal proteins may be potential targets for early diagnosis of MDD.

Moreover, our current studies found that all of the top 20 hub genes were identified as being downregulated in MDD. We speculated that the reduced expression of NSA2 and the other ribosomal genes might play an important role in MDD, and these identified genes may be potential therapeutic targets for MDD. Further analyses were necessary to analyze the effect of these gene agonists on MDD and verify the mechanisms underlying the target gene agonist-induced improvements by evaluating the gene expression profile, the histological and biochemical parameters, and behavioral tests in MDD animal models and MDD patients.

Our study has a few limitations. First, to comprehensively identify the dysfunctions in MDD, integrated analysis of both venous blood samples and brain tissues is warranted; this was not performed in the present study. Second, in order to determine the diagnostic accuracy of the hub genes associated with MDD, it will be helpful to increase the sample size for further external validation. Third, single microarray analysis may contribute to high false-positive rate and one-sided results; thus, it is necessary to improve detection power by integrating multiple individual data in a future study. Fourth, due to the heterogeneity of depression and the lack of clinical data, we were unable to evaluate the associations between risk indicators and stratification of patients based on the severity of MDD. Fifth, not all depressed patients have mitochondrial dysfunction and inflammation. It is possible that these alterations are present only in specific subgroups of depressed patients, with specific clinical and pathophysiological features. For example, increased inflammation is identified in a subgroup of MDD patients who have a neurodevelopmental form of depression, deriving from exposure to stress early in childhood or in utero (Miller & Raison, 2016; Pariante, 2017). More clinical and demographic characteristics of MDD patients is needed to be included in the study for further subgroup analysis. Finally, further experimental evidence, such as real-time PCR, western blot, immunohistochemistry assays, is required to fully elucidate the role of hub genes and the underlying mechanisms of MDD.

These data suggest multiple pathways and biomarkers of MDD, consistent with our current knowledge of the pathophysiology of this disease. We believe that this hypothesis-generating study provides new insight into the molecular mechanisms underlying MDD, identifying several potential biomarkers for its diagnosis and treatment.

## Conclusions

In conclusions, the aim of this study was to explore the molecular mechanisms underlying the progression of MDD via a comprehensive bioinformatics analysis that aimed to identify the associated biological functions and pathways involved in the development of MDD. Moreover, we also identified 20 candidate genes which could serve as potential diagnostic biomarkers through PPI network analysis, the functional similarity analysis, and ROC curve analysis. However, more molecular experiments are needed for further validation of the findings of current study.

## Supplemental Information

10.7717/peerj.7171/supp-1 Supplemental Information 1 All differently expressed genes in Venn diagram. Venn diagram of the number of DEGs identified as significant for the cross-validated comparisons.

10.7717/peerj.7171/supp-2 Supplemental Information 2 Hub genes in major depressive disorder-specific network. The hub genes with the top 20 scores were analyzed using the NetworkAnalyzer plugin.

## Additional Information and Declarations

### Microarray Data Deposition

The following information was supplied regarding the deposition of microarray data: This work used a publicly available dataset, available at NCBI: GSE98793.

### Data Availability

The following information was supplied regarding data availability: The raw measurements are available in the Supplemental Files as Tables S1 and S2.
